# Colon cancer metastasis to mediastinal lymph nodes without liver or lung involvement: A case report

**DOI:** 10.3892/ol.2014.2426

**Published:** 2014-08-08

**Authors:** MUSTAPHA M. EL-HALABI, SAID A. CHAABAN, JOSEPH MEOUCHY, SETH PAGE, WILLIAM J. SALYERS

**Affiliations:** 1 Department of Internal Medicine, University of Kansas School of Medicine, Wichita, KS 67214, USA; 2 Cancer Center of Kansas, Wichita, KS 67214, USA

**Keywords:** colorectal, carcinoma, lymphadenopathy, distant, mediastinum

## Abstract

Colon cancer is the second most common type of cancer in females and the third in males, worldwide. The most common sites of colon cancer metastasis are the regional lymph nodes, liver, lung, bone and brain. In this study, an extremely rare case of colon adenocarcinoma with extensive metastasis to the mediastinal lymph nodes without any other organ involvement is presented. A 44-year-old Caucasian male presented with abdominal pain, a change in bowel habits, melena and weight loss. Colonoscopy revealed a large friable, ulcerated, circumferential mass in the ascending colon. Biopsies were consistent with the diagnosis of invasive moderately differentiated adenocarcinoma. Subsequently, right colon resection was performed, and pathological analysis revealed moderately differentiated adenocarcinoma of the right colon with extensive regional lymph node involvement. Computed tomography (CT) scans of the chest, abdomen and pelvis were performed preoperatively as part of routine staging for colon cancer. No liver or lung pathology was identified; however, multiple pathologically enlarged mediastinal lymph nodes were observed. Endoscopic ultrasound with fine needle aspiration of the largest mediastinal lymph node, which measured 5.2×3.5 cm on CT scans, was performed. The pathology was again consistent with the diagnosis of metastatic colorectal primary adenocarcinoma. At present, no optimum treatment has been identified for metastatic colon cancer to the mediastinal lymph nodes. The patient in the current case received chemotherapy with folinic acid, fluorouracil and oxaliplatin (FOLFOX), as well as with bevacizumab. Initial follow-up CT scans of the chest revealed a positive response to treatment. Physicians, in particular, radiologists, must consider the mediastinum during the first evaluation and further follow-up of patients with colorectal carcinoma even in the absence of metastasis.

## Introduction

Colorectal cancer (CRC) is the second most common type of cancer in females and the third in males, worldwide (1). It is also the second most common cause of cancer-related mortality in the United States (2). Approximately one third of patients who develop CRC succumb to the disease (2). CRC is diagnosed in the majority of patients following the onset of symptoms which include rectal bleeding, a change in bowel habits, bowel obstruction and weight loss, or following the identification of occult bleeding (3,4). However, the implementation of CRC screening guidelines has improved the detection of pre-malignant polyps and early-stage asymptomatic CRC, and thus has improved disease outcomes (3,4). The most prevalent sites of metastasis are the regional lymph nodes, liver, lung, bone and brain (5). In the present study, an extremely rare case of colon adenocarcinoma with extensive metastasis to multiple mediastinal lymph nodes without any other organ involvement is presented. The patient provided written informed consent for the publication of this study.

## Case report

### Patient presentation

**Figure 1:**
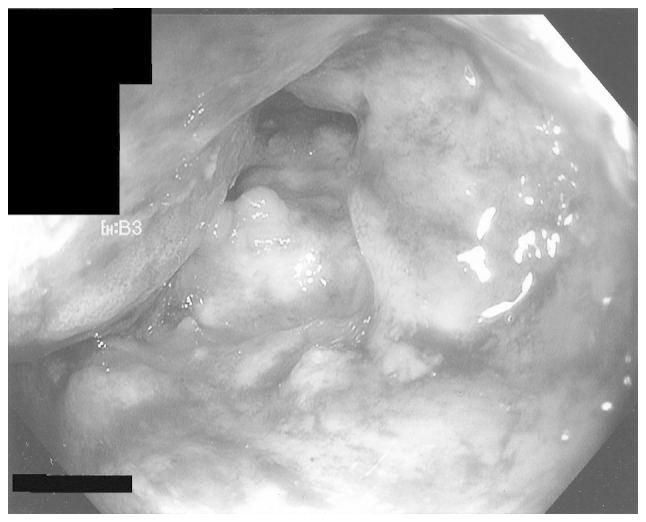
Colonoscopy revealing an ascending colon mass.

This study presents the case of a 44 year-old Caucasian male with a one-year history of waxing and waning right-sided abdominal pain associated with a change in bowel habits, melena and a weight loss of ~9 kg. Therefore, colonoscopy was performed, which revealed a large, friable, ulcerated, circumferential mass in the ascending colon, which was almost completely obstructing the ascending colon and was unable to be safely traversed with the colonoscope (Fig. 1). Biopsies were consistent with the diagnosis of invasive moderately differentiated adenocarcinoma. The level of carcinoembryonic antigen (CEA) in the patient’s blood was found to be 3.0 ng/ml (normal range, <2.5 ng/ml).

### Cancer staging

**Figure 2:**
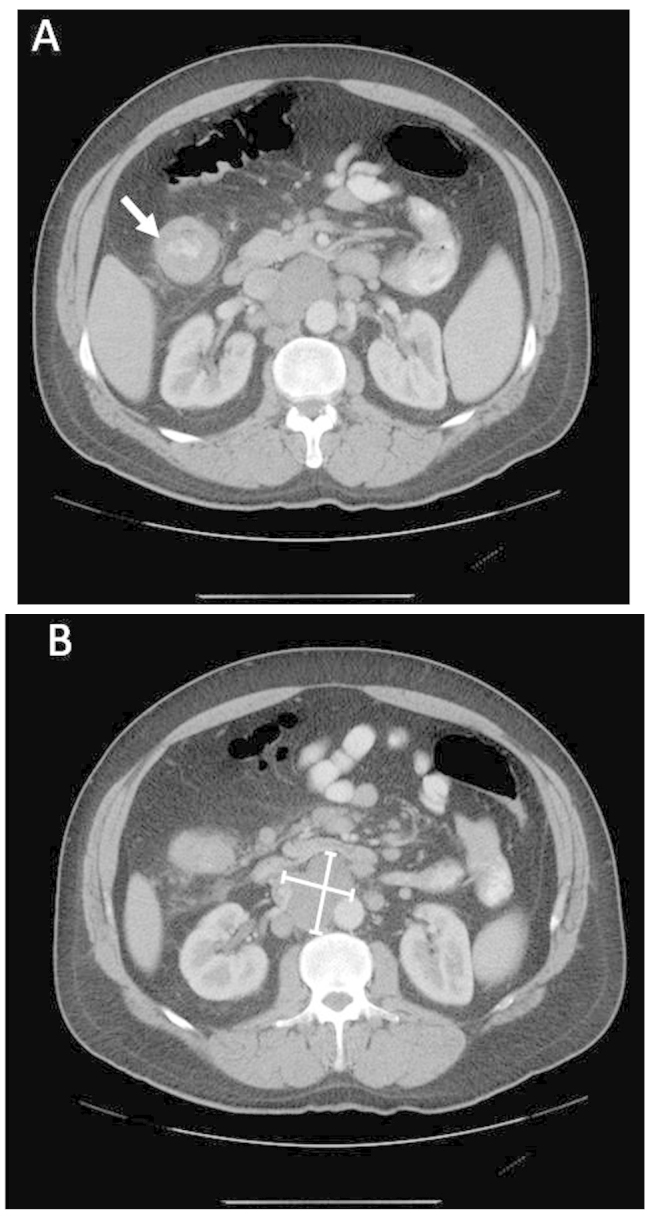
Computed tomography scan of the abdomen showing (A) the proximal colon mass and (B) the large peri-aortic lymph nodes.

**Figure 3:**
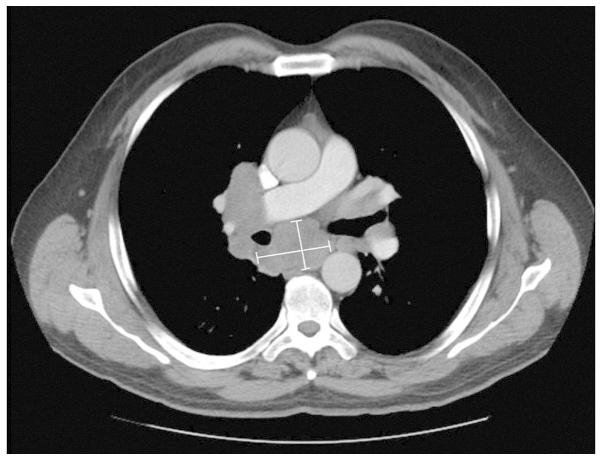
Computed tomography scan of the chest showing the large subcarinal and precarinal lymph nodes. White lines present the largest dimensions of the subcarinal lymph node observed (5.2×3.5 cm).

Computed tomography (CT) scans of the chest, abdomen and pelvis were performed as part of the staging process of colon cancer. Abdominal images revealed a large concentric mass involving the ascending colon that extended ~7 cm in length with marked narrowing of the colon, without causing complete obstruction (Fig. 2). Retroperitoneal and mesenteric lymphadenopathy were also observed. No liver pathology was identified by CT. The CT scan of the chest revealed multiple pathologically enlarged mediastinal lymph nodes with the largest sizes exhibited by the pre-aortic lymph node (3.0×2.9 cm), a precarinal lymph node (3.6×4.9 cm), a subcarinal lymph node (5.2×3.5 cm) and a right hilar lymph node (3.3×5.2 cm), in addition to bilateral smaller hilar lymph nodes (Fig. 3). No metastasis or other pathologies was identified inside the lungs by CT.

### Mediastinal lymph node involvement

**Figure 4:**
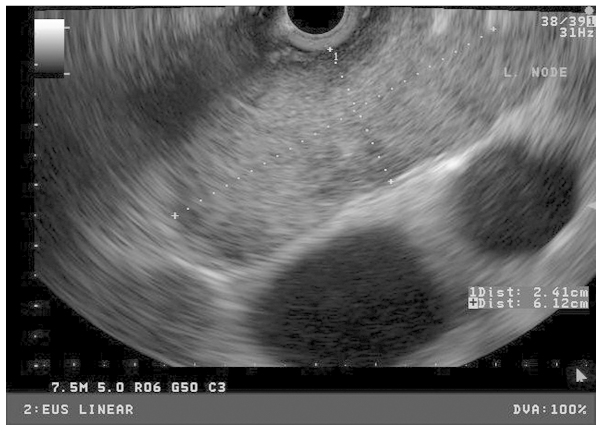
Endoscopic ultrasound of the largest mediastinal lymph node, located in the subcarinal region, measuring 2.41×6.12 cm in diameter.

**Figure 5:**
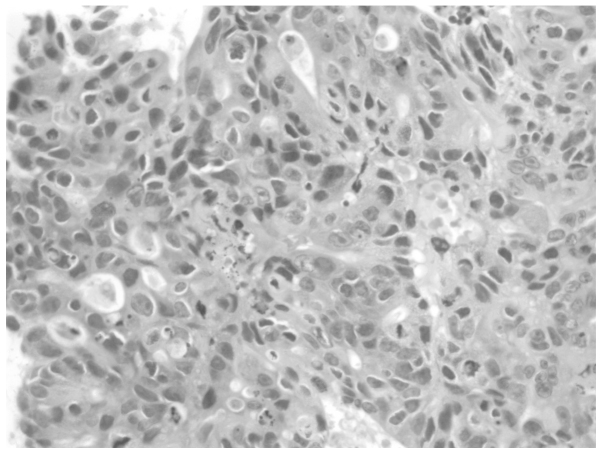
Core biopsy of the mediastinal lymph node showing adenocarcinoma (stain, hematoxylin and eosin; magnification, ×40).

Endoscopic ultrasound (EUS) with fine-needle aspiration was performed to exclude lymphoma. EUS did not reveal any celiac lymphadenopathy; however, multiple enlarged mediastinal lymph nodes were identified and the largest was located in the subcarinal region (2.41×6.12 cm) (Fig. 4). The pathology was again consistent with the diagnosis of metastatic adenocarcinoma (Fig. 5) and immunohistochemical staining of the core biopsy samples revealed a cytokeratin 7 (CK7)-negative/CK20-positive pattern, which is typical of colorectal primary carcinoma. Prior to receiving the pathology results of the mediastinal lymph node biopsies, the patient developed symptoms of colonic obstruction. The patient was managed conservatively with bowel rest, and continuous suctioning of the gastric contents via a nasogastric tube. Obstructive symptoms were resolved after two days.

### Colon resection

Following the pathological examination of the lymph nodes the patient underwent right colon resection from the terminal ileum proximally to the mid transverse colon distally. Pathology results revealed moderately differentiated adenocarcinoma of the right colon (largest tumor dimension, 7 cm), demonstrating invasion of the muscularis propria with extensive infiltration of pericolic fat and discontinuous extramural extension, prominent and extensive lymph-vascular invasion, and metastatic involvement of 33 out of 52 regional lymph nodes. The circumferential (radial) resection margin was positive and the proximal and distal margins were negative.

### Chemotherapy and follow-up

The patient began chemotherapy treatment, and was treated with eight cycles of folinic acid, fluorouracil and oxaliplatin plus Avastin prior to developing peripheral neuropathy. Therefore, the patient was administered infusional fluorouracil and Avastin. The patient is alive one year following hemicolectomy. Initial follow-up CT scans of the chest revealed a positive response to chemotherapy with certain shrinkage of the mediastinal lymph nodes after a small number of cycles. Nine months following the diagnosis of CRC, CT scans revealed a stable disease and CEA levels were observed to be 3.7 ng/ml.

## Discussion

In summary, the patient exhibited a proximal colon adenocarcinoma with extensive regional (retroperitoneal and mesenteric) lymphadenopathy and distal metastasis to the mediastinal lymph nodes without liver, lung or any other organ involvement. This case demonstrated an extremely rare pattern of colon cancer metastasis (5). It is hypothesized that mediastinal involvement is usually a re-metastasis from a previously metastasized site, such as the lung or liver. The re-metastasis of colon cancer to the mediastinal lymph nodes from lung metastasis is rare; however, it has been reported in a small number of studies (6). Re-metastasis from the liver to lymph nodes draining the liver is even rarer, but has also been reported in the literature (7). Rashidi *et al* (8) demonstrated, using an orthotopic mouse model, that re-metastasis from the liver to all of its draining lymphatic systems, including the portal, celiac and mediastinal lymph nodes, is possible (8). However, re-metastasis from the liver to the mediastinal lymph nodes in humans is extremely rare and has been reported in only four cases in the literature (7,9–11). Ovarian re-metastasis to the mediastinal lymph nodes also appears to be possible; however, only a single case report exists that describes this (12). Re-metastasis to the mediastinal lymph nodes has also been reported in a single patient with brain metastasis (13). However, direct colon metastasis to the mediastinal lymph nodes without liver, lung or any other organ involvement has been reported in a single case report by Musallam *et al* (14), who demonstrated solitary mediastinal lymph node involvement, in which only one lymph node was involved. However, to the best of our knowledge, the present study is the first to identify extensive metastasis of colorectal adenocarcinoma to multiple mediastinal lymph nodes without any other organ or site involvement. The exact route of this metastasis is unclear. However, we hypothesize that involvement of the mediastinal lymph nodes was via the para-aortic lymphatic drainage route, or via ‘skip’ metastasis, as the patient exhibited retroperitoneal peri-aortic lymph node involvement.

The optimal treatment option for metastatic colon cancer to mediastinal lymph nodes is unclear, given the scarcity of cases reported in the literature. Previous studies have reported surgical resection of a solitary mediastinal lymph node with positive outcomes (11), and systemic treatment with chemotherapy with favorable outcomes (14). However, in the present case, due to multiple lymph node involvement being observed, the lymph nodes were not surgically resected and chemotherapy was administered instead. At present, the response appears favorable, as the patient is alive one year following diagnosis. In conclusion, physicians, in particular radiologists, must consider the mediastinum during the first evaluation and further follow-up of patients with colorectal carcinoma even in the absence of metastasis.
